# Assessment of Eustachian tube function in patients with tympanic membrane retraction and in normal subjects^☆^^☆☆^

**DOI:** 10.1016/j.bjorl.2016.01.010

**Published:** 2016-04-25

**Authors:** Inesângela Canali, Letícia Petersen Schmidt Rosito, Bruno Siliprandi, Cláudia Giugno, Sady Selaimen da Costa

**Affiliations:** a Universidade Federal do Rio Grande do Sul (UFRGS), Porto Alegre, RS, Brazil; b Universidade Federal do Rio Grande do Sul (UFRGS), Departamento de Oftalmologia e Otorrinolaringologia, Porto Alegre, RS, Brazil

**Keywords:** Eustachian tube/physiopathology, Ear disease/physiopathology, Middle ear ventilation/methods, Valsalva maneuver/physiology, Analysis of variance, Child, Tuba de Eustáquio/fisiopatologia, Doença otológica/fisiopatologia, Ventilação do ouvido médio/métodos, Manobra de Valsalva/fisiologia, Análise de variância, Crianças

## Abstract

**Introduction:**

The diagnosis of Eustachian tube dysfunctions is essential for better understanding of the pathogenesis of chronic otitis media. A series of tests to assess tube function are described in the literature; however, they are methodologically heterogeneous, with differences ranging from application protocols to standardization of tests and their results.

**Objective:**

To evaluate the variation in middle ear pressure in patients with tympanic membrane retraction and in normal patients during tube function tests, as well as to evaluate intra-individual variation between these tests.

**Methods:**

An observational, contemporary, cross-sectional study was conducted, in which the factor under study was the variation in middle ear pressure during tube function tests (Valsalva maneuver, sniff test, Toynbee maneuver) in healthy patients and in patients with mild and moderate/severe tympanic retraction. A total of 38 patients (76 ears) were included in the study. Patients underwent tube function tests at two different time points to determine pressure measurements after each maneuver. Statistical analysis was performed using SPSS software, version 18.0, considering *p*-values <0.05 as statistically significant.

**Results:**

Mean (standard deviation) age was 11 (2.72) years; 55.3% of patients were male and 44.7% female. The prevalence of type A tympanogram was higher among participants with healthy ears and those with mild retraction, whereas type C tympanograms were more frequent in the moderate/severe retraction group. An increase in middle ear pressure was observed during the Valsalva maneuver at the first time point evaluated in all three groups of ears (*p* = 0.012). The variation in pressure was not significant either for the sniff test or for the Toynbee maneuver at the two time points evaluated (*p* ≥ 0.05). Agreement between measurements obtained at the two different time points was weak to moderate for all tests in all three groups of ears, and the variations in discrepancy between measurements were higher in ears with moderate/severe tympanic retraction.

**Conclusion:**

In this study population, the mean pressure in the middle ear showed significant variation only during the Valsalva maneuver at the first time point evaluated in the three groups of ears. Normal ears and those with mild retraction behaved similarly in all tests. The tested maneuvers exhibited weak to moderate intra-individual variation, with the greatest variation occurring in ears with moderate/severe retraction.

## Resumo

**Introdução:**

O diagnóstico das disfunções da tuba auditiva é essencial para o melhor entendimento da patogênese da otite média crônica. A literatura descreve uma série de testes que avaliam a função tubária; contudo, tais exames são metodologicamente heterogêneos, com diferenças que variam desde os protocolos de aplicação até a padronização dos exames e seus resultados.

**Objetivo:**

Avaliar a variação na pressão na orelha média em pacientes com retração da membrana timpânica e em indivíduos normais durante a realização dos testes de função tubária, e também avaliar a variação intraindividual desses testes.

**Método:**

Estudo observacional do tipo transversal e contemporâneo, no qual o fator em estudo foi a variação na pressão na orelha média durante a realização dos testes de função tubária (manobra de Valsalva, *Sniff Test* e manobra de Toynbee) em indivíduos normais e em pacientes com retrações timpânicas leves e moderadas/graves. Foram incluídos 38 pacientes (76 orelhas). Os pacientes foram submetidos, em dois momentos diferentes, a testes de função tubária para determinar as medidas de pressão após cada manobra. A análise estatística foi realizada com o uso do programa SPSS, versão 18.0, e consideramos como estatisticamente significativos os valores de *p* < 0,05.

**Resultados:**

A média ± desvio padrão da idade foi de 11 ± 2,72 anos; 55,3% dos pacientes eram do gênero masculino e 44,7% pertenciam ao gênero feminino. A prevalência de curvas timpanométricas do tipo A foi mais alta entre os participantes com orelhas normais e naqueles com retrações leves, enquanto as curvas timpanométricas do tipo C foram mais frequentes no grupo com retrações moderadas/graves. Observamos pressões aumentadas na orelha média durante a realização da manobra de Valsalva no primeiro momento da avaliação nos três grupos de orelhas (*p* = 0,012). A variação na pressão não foi significativa para o *Sniff Test*, nem para a manobra de Toynbee nos dois momentos de avaliação (*p* ≥ 0,05). Consideramos que a concordância entre as determinações obtidas nos dois momentos diferentes foi fraca a moderada para todos os testes nos três grupos de orelhas, e as variações em termos de discrepância entre as medidas foram maiores nas orelhas com retrações timpânicas moderadas/graves.

**Conclusão:**

Na população estudada, a média das pressões na orelha média apresentou variação significante apenas durante a manobra de Valsalva no primeiro momento de avaliação, nos três grupos de orelhas. As orelhas normais e aquelas apresentando retração leve se comportaram de maneira similar nos testes realizados. As manobras testadas exibiram uma variação intraindividual fraca a moderada, e a maior variação ocorreu nas orelhas com retrações moderadas/graves.

## Introduction

The Eustachian tube (ET), or auditory tube, is the main structure responsible for equalizing pressure between the middle ear and the outside environment, ensuring ventilation of the air spaces of the temporal bone, and protecting the middle ear from nasopharyngeal secretions.^1^ Persistent ET dysfunction may produce negative pressure within the tympanic cavity, resulting in a shift of intravascular fluid into the interstitial spaces and then into the middle ear lumen,^2^ or causing retraction of the tympanic membrane (TM), and is thus one of the earliest landmarks of the pathogenesis of chronic otitis media (COM).^3^, ^4^ Therefore, several studies have pinpointed ET dysfunction as one of the factors for perpetuation of otitis media with effusion (OME), progression of OME to moderate and severe TM retraction, and progression of the latter to chronic cholesteatomatous otitis media.

The diagnosis of ET dysfunction is therefore essential for a better understanding of COM pathogenesis. Several tests of ET function have been described in the literature.^5^, ^6^, ^7^ However, these tests are methodologically heterogeneous in aspects ranging from application protocols to standardization of tests and their results. Hence, the true applicability of these tests is a matter of debate, particularly in patients with questionable ET patency. The Eustachian tube function (ETF) tests most commonly employed in patients with intact TM include the Valsalva maneuver, the sniff test, and the Toynbee maneuver.^1^, ^8^, ^9^

The present study sought to assess variation in middle ear pressure in patients with mild and moderate/severe TM retraction and healthy patients during ETF tests (Valsalva maneuver, sniff test, and Toynbee maneuver), as well as assess intra-individual variation in these tests in the three aforementioned patient groups.

## Methods

This was an observational, cross-sectional, contemporary study. The factor under study was variation in pressure within the middle ear during ETF testing in patients with mild TM retraction, moderate/severe TM retraction, or healthy TMs. The sample comprised 38 patients aged 8–18 years, recruited from the outpatient otolaryngology clinic of a tertiary care center from December 1, 2012, to March 31, 2013. Patients were allocated into three groups according to the severity of pars tensa retraction in the worse ear, using the modified Sadé and Berco (1976) classification proposed by Costa et al., as follows: group 1 – patients with normal TMs bilaterally (controls); group 2 – patients with mild TM retraction in at least one ear; and group 3 – patients with moderate or severe TM retraction in at least one ear.

Inclusion criteria for the patient group were: age between 8 and 18 years; mild, moderate, or severe TM retraction in at least one ear; and intact TMs in both ears. The inclusion criteria for controls were: good overall health; same age range as participants in the patient group; current outpatient follow-up for adenoidectomy or adenotonsillectomy performed at least six months before recruitment; and normal TMs bilaterally. Exclusion criteria for patients and controls were: middle ear effusion; cleft lip and palate or other craniofacial abnormalities; Down syndrome; mucopolysaccharidoses; immunosuppression-related diseases; nasal or nasopharyngeal obstruction; inability to undergo audiometry, aural toilet, video otoscopy, or ETF tests; and refusal to participate.

All patients underwent a thorough, targeted interview during the first study visit. Video otoscopy was performed and recorded. Findings were systematically described by a senior otologist using a dedicated form. All patients also underwent nasal endoscopy for assessment of nasopharyngeal obstruction. Pure tone and speech audiometry was also performed during first assessment of all patients and controls.

Assessment of ETF consisted of the following tests, which were performed with the Interacoustics AZ26 and AT235h impedance audiometers to measure middle ear pressure after each test maneuver. First, tympanometry was performed in both ears to record the baseline pressure in each ear prior to testing and ascertain its type of tympanogram, according to the Jerger (1970) classification. This was followed by the Valsalva maneuver, sniff test, and Toynbee maneuver, which were performed sequentially, first in the right ear and then in the left. During each test, middle ear pressure was measured five times consecutively as described below:

*Valsalva maneuver*: the patient was asked to perform five consecutive Valsalva maneuvers. Middle ear pressure was measured and recorded immediately after each maneuver (VP1–VP5), during which time the patient was asked to refrain from speaking or swallowing.
*Sniff test*: the patient was asked to inhale forcefully through the nose (mouth closed) five times consecutively. Again, middle ear pressure was measured and recorded immediately after each maneuver (SP1–SP5), during which time the patient was asked to refrain from speaking or swallowing.
*Toynbee maneuver*: the patient was asked to swallow a sip of water while his or her nose was pinched shut by the investigator, five times consecutively. Middle ear pressure was measured and recorded immediately after each maneuver (TP1–TP5), during which time the patient was asked to refrain from speaking or swallowing.

Between each test, a 5-minute interval was enforced and the patient was instructed to drink water, in an attempt to return pressure to baseline values. Baseline pressure before each test was recorded as well (baseline pressure before Valsalva maneuver [VBP]; baseline pressure before sniff test [SBP]; and baseline pressure before Toynbee test [TBP]).

All of the aforementioned tests were performed at a second time point of assessment, 15–30 days after the first study visit. Results were described as corresponding to the first or second time point of assessment.

To detect a difference in ETF measured by means of a quantitative variable with approximately normal distribution in the three study groups, with a statistical power of 80% and a significance level of *α* = 0.05, the minimum sample size was calculated as 12 controls and 24 patients with TM retraction (12 mild and 12 moderate/severe). Data were stored in a dedicated database in Excel. SPSS v. 18.0 for Windows was used for statistical analyses. Quantitative data were expressed as means and standard deviations, and categorical data, as absolute and relative frequencies. Analysis of middle ear pressure measurements was based on mixed-effects (fixed and random) models, taking into account intra-subject correlated observations, both for ears and for repeated measures. Categorical data were analyzed by means of a generalized estimating equations (GEE) model. The Bland–Altman method and intraclass correlation coefficients were used for assessment of agreement between measurements.

This study was approved by the ethics committee of the Group of Research and Graduate Studies under No. 12-0432. An informed consent was signed for the anonymous use of patient data by the legal guardians of all participants. Treatment was not affected in any way whether patients provided or refused informed consent. As this study also used historical data for analysis, all authors signed an agreement for the confidential use of data.

## Results

A total of 38 participants were assessed: 14 healthy controls, 12 patients with mild TM retraction in at least one ear, and 12 patients with moderate/severe TM retraction in at least one ear. Separate analysis of each ear revealed 36 healthy ears, 23 ears with mild retraction, and 17 ears with moderate/severe retraction. Mean age and standard deviation (SD) was 11 (2.72) years (range, 8–17 years). Patient distribution according to sex was 55.3% male and 44.7% female.

The prevalence of type A tympanogram was higher in groups 1 and 2, whereas type C tympanograms were most common in group 3, at both time points of assessment. There were significant differences among the three groups at the first and second time points of assessment (*p* = 0.002 and *p* < 0.001, respectively, chi-squared test).

### Eustachian tube function tests

#### Valsalva maneuver

**Figure 1:**
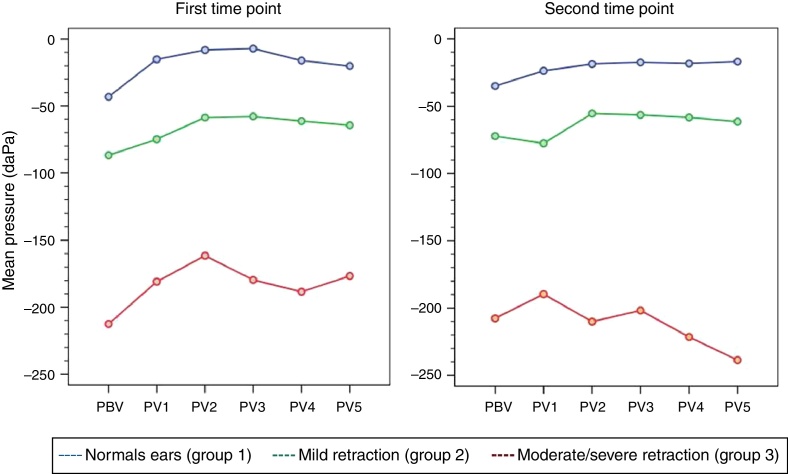
Variability of mean middle ear pressure measurements during Valsalva maneuver at the first and second time points of assessment.

At the first time point of assessment with the Valsalva maneuver, there was a trend toward increasing middle ear pressures from baseline (VBP) during each of the five consecutive Valsalva maneuvers (VP1–VP5) in all three groups of ears, with *p* = 0.012 (*p* [measurement]). Mean pressures (VP1–VP5) were different in each group, with *p* < 0.001 (*p* [group]). At the second time point of assessment, the trend toward pressures increasing from VBP during each of the five consecutive maneuvers (VP1–VP5) remained; however, due to the behavior of pressure measurements in group 3, *p*-values did not reach statistical significance (*p* [measurement] = 0.707). Again, mean pressures (VP1–VP5) were different in each group, as at the first time point of assessment (*p* [group] < 0.001). Table 1, Table 2 show the estimated mean pressures at baseline and after five consecutive Valsalva maneuvers, after mixed-models adjustment, in the three study groups, at the first and second time points of assessment, respectively. At both time points, the peak increase in middle ear pressure occurred during the first three maneuvers, in all three study groups. Fig. 1 shows the variability in mean pressures in the three study groups over the course of the test maneuver process (VBP and VP1–VP5), at the first and second time points of assessment.

**Table 1:** Estimated means obtained with a linear mixed model for the Valsalva maneuver in the three groups, at the first time point of assessment.

Measurement	Normal ears (*n* = 36)	Mild retraction (*n* = 23)	Moderate/severe retraction (*n* = 17)
VBP	−51 (±17)	−85 (±19)	−199 (±21)
VP1	−23 (±17)	−73 (±19)	−167 (±21)
VP2	−16 (±17)	−57 (±19)	−148 (±21)
VP3	−15 (±17)	−56 (±19)	−166 (±21)
VP4	−24 (±17)	−59 (±19)	−175 (±21)
VP5	−28 (±17)	−63 (±19)	−163 (±21)

VBP, Valsalva maneuver baseline pressure; VP, Valsalva maneuver pressure.

Data expressed as mean (standard error), (*p* [measurement]) = 0.012; (*p* [group]) < 0.001.

**Table 2:** Estimated means obtained with a linear mixed model for the Valsalva maneuver in the three groups, at the second time point of assessment.

Measurement	Normal ears (*n* = 36)	Mild retraction (*n* = 23)	Moderate/severe retraction (*n* = 17)
VBP	−53 (±18)	−84 (±20)	−154 (±22)
VP1	−42 (±18)	−89 (±20)	−136 (±22)
VP2	−37 (±18)	−67 (±20)	−156 (±22)
VP3	−36 (±18)	−68 (±20)	−148 (±22)
VP4	−37 (±18)	−70 (±20)	−168 (±22)
VP5	−36 (±18)	−73 (±20)	−185 (±22)

VBP, Valsalva maneuver baseline pressure; VP, Valsalva maneuver pressure.

Data expressed as mean (standard error); (*p* [measurement]) = 0.707; (*p* [group]) < 0.001.

#### Sniff test

**Figure 2:**
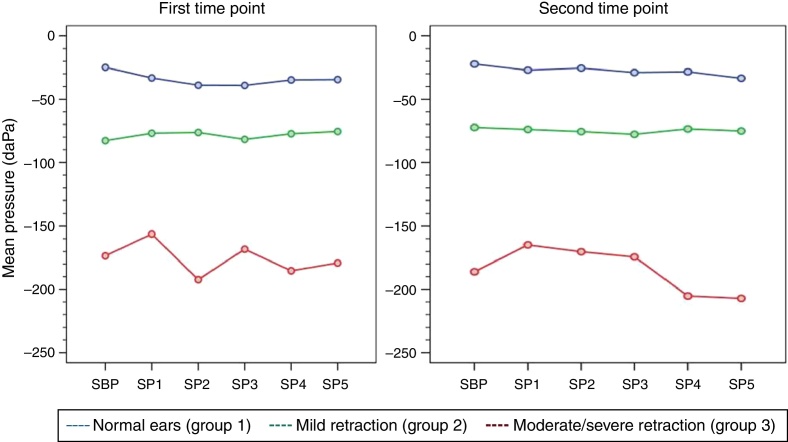
Variability of mean middle ear pressure measurements during the sniff test at the first and second time points of assessment.

At both time points of assessment with the sniff test, there was no trend toward decreases in middle ear pressures from baseline (SBP) during each of the five consecutive test maneuvers (SP1–SP5) in any of the three groups of ears, as represented by the *p*-values obtained at the first and second time points of assessment (*p* [measurement] = 0.716, *p* [measurement] = 0.477, respectively). Mean pressures (SP1–SP5) were different in each group at both time points (*p* < 0.001). Table 3, Table 4 show the estimated mean pressures at baseline and after five consecutive test maneuvers, after mixed-models adjustment, in the three study groups, at the first and second time points of assessment, respectively. Fig. 2 illustrates the variability in mean pressures, in the three study groups, over the course of the test maneuver process (SBP and SP1–SP5) at the first and second time points of assessment.

**Table 3:** Estimated means obtained with a linear mixed model for the sniff test in the three study groups, at the first time point of assessment.

Measurement	Normal ears (*n* = 36)	Mild retraction (*n* = 23)	Moderate/severe retraction (*n* = 17)
SBP	−31 (±15)	−85 (±16)	−157 (±18)
SP1	−40 (±15)	−79 (±16)	−140 (±18)
SP2	−45 (±15)	−78 (±16)	−176 (±18)
SP3	−46 (±15)	−84 (±16)	−152 (±18)
SP4	−41 (±15)	−79 (±16)	−169 (±18)
SP5	−41 (±15)	−77 (±16)	−163 (±18)

SBP, sniff test baseline pressure; SP, sniff test pressure.

Data expressed as mean (standard error); (*p* [measurement]) = 0.716; (*p* [group]) < 0.001.

**Table 4:** Estimated means obtained with a linear mixed model for the sniff test in the three study groups, at the second time point of assessment.

Measurement	Normal ears (*n* = 36)	Mild retraction (*n* = 23)	Moderate/severe retraction (*n* = 17)
SBP	−42 (±17)	−71 (±19)	−146 (±21)
SP1	−47 (±17)	−72 (±19)	−124 (±21)
SP2	−46 (±17)	−74 (±19)	−130 (±21)
SP3	−49 (±17)	−76 (±19)	−134 (±21)
SP4	−49 (±17)	−72 (±19)	−165 (±21)
SP5	−54 (±17)	−73 (±19)	−167 (±21)

SBP, sniff test baseline pressure; SP, sniff test pressure.

Data expressed as mean (standard error); (*p* [measurement]) = 0.477; (*p* [group]) < 0.001.

#### Toynbee maneuver

**Figure 3:**
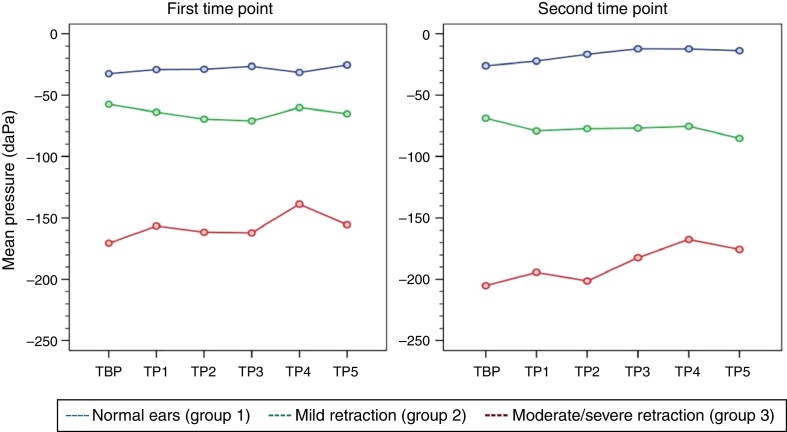
Variability of mean middle ear pressure measurements during the Toynbee maneuver at the first and second time points of assessment.

At both time points of assessment with the Toynbee maneuver, there were no trends toward either decrease or increase in middle ear pressures from baseline (TBP) during each of the five consecutive test maneuvers (TP1–TP5) in any of the three groups of ears, as represented by the *p*-values obtained at the first and second time points of assessment (*p* [measurement] = 0.945 and 0.440, respectively). Mean pressures (TP1–TP5) were different in each group at both time points (*p* < 0.001). Table 5, Table 6 show the estimated mean pressures at baseline and after five consecutive test maneuvers after mixed-models adjustment, in the three study groups, at the first and second time points of assessment, respectively. Fig. 3 demonstrates the variability in mean pressures in the three study groups over the course of the test maneuver process (TBP and TP1–TP5), at the first and second time points of assessment.

**Table 5:** Estimated means obtained with a linear mixed model for the Toynbee maneuver in the three study groups, at the first time point of assessment.

Measurement	Normal ears (*n* = 36)	Mild retraction (*n* = 23)	Moderate/severe retraction (*n* = 17)
TBP	−50 (±16)	−44 (±18)	−152 (±20)
TP1	−47 (±16)	−51 (±18)	−138 (±20)
TP2	−46 (±16)	−56 (±18)	−143 (±20)
TP3	−44 (±16)	−58 (±18)	−143 (±20)
TP4	−49 (±16)	−47 (±18)	−124 (±20)
TP5	−43 (±16)	−52 (±18)	−141 (±20)

TBP, Toynbee maneuver baseline pressure; TP, Toynbee maneuver pressure.

Data expressed as mean (standard error); (*p* [measurement]) = 0.945; (*p* [group]) < 0.001.

**Table 6:** Estimated means obtained with a linear mixed model for the Toynbee maneuver in the three study groups, at the second time point of assessment.

Measurement	Normal ears (*n* = 36)	Mild retraction (*n* = 23)	Moderate/severe retraction (*n* = 17)
TBP	−54 (±16)	−67 (±18)	−148 (±20)
TP1	−50 (±16)	−78 (±18)	−137 (±20)
TP2	−45 (±16)	−76 (±18)	−144 (±20)
TP3	−40 (±16)	−75 (±18)	−125 (±20)
TP4	−40 (±16)	−74 (±18)	−110 (±20)
TP5	−42 (±16)	−84 (±18)	−118 (±20)

TBP, Toynbee maneuver baseline pressure; TP, Toynbee maneuver pressure.

Data expressed as mean (standard error); (*p* [measurement]) = 0.440; (*p* [group]) < 0.001.

To assess the degree of agreement between the two time points of middle ear pressure measurement during the performance of ETF tests, scatter charts were plotted for each test and intraclass correlation coefficients (ICCs) were calculated. The ICCs were 0.65 for the Valsalva maneuver, 0.67 for the sniff test, and 0.63 for the Toynbee maneuver, which correspond to moderate agreement between the two time points of assessment across all three tests. Bland–Altman plots of agreement between middle ear pressures at the two time points of assessment showed poor replicability of results with all three test maneuvers.

**Figure 4:**
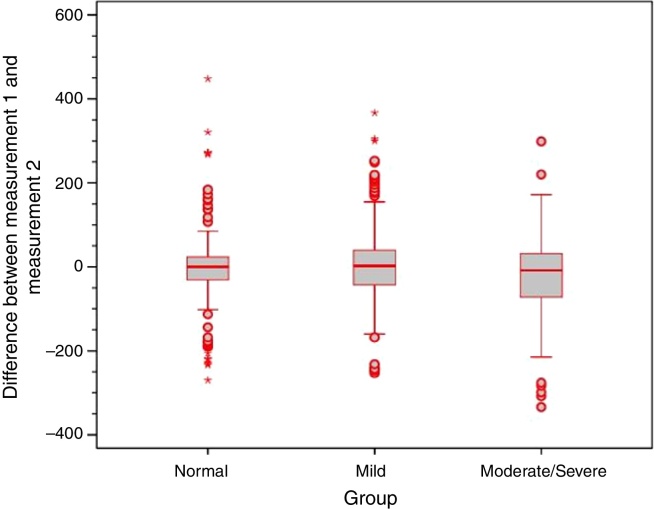
Box plot of differences between time points 1 and 2 for the Valsalva maneuver in the three study groups.

**Figure 5:**
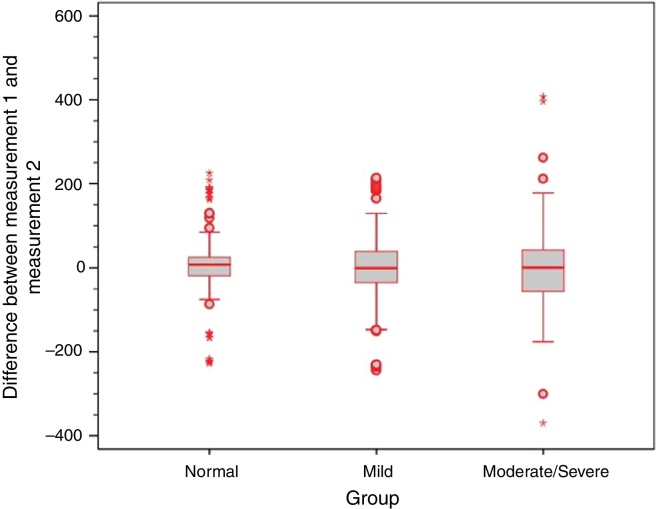
Box plot of differences between time points 1 and 2 for the sniff test in the three study groups.

**Figure 6:**
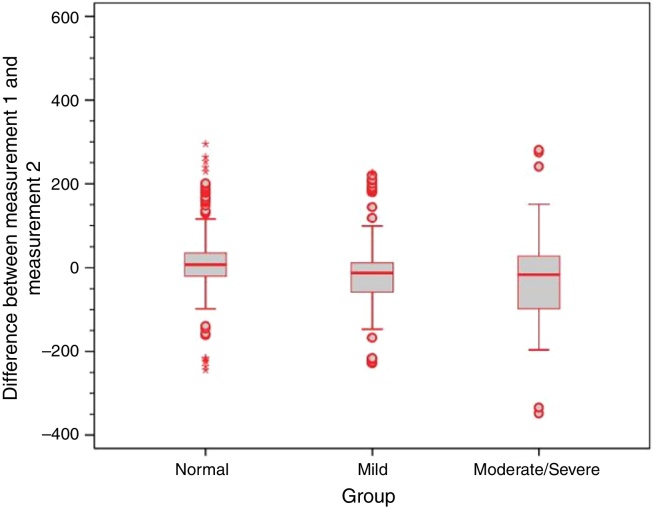
Box plot of differences between time points 1 and 2 for the Toynbee maneuver in the three study groups.

Analysis of the error variance between measurements obtained at the first and second time points of assessment in the three study groups yielded statistically significant results (*p* = 0.018 for Valsalva maneuver; *p* < 0.001 for sniff test; and *p* = 0.005 for Toynbee maneuver). Apparently, the variances in the discrepancies between measurements obtained at the first and second time points of assessment were greater in the moderate/severe TM retraction group across all three ETF tests, as shown by the box plots below (Figure 4, Figure 5, Figure 6).

## Discussion

Eustachian tube function has been the subject of many clinical and experimental studies, most seeking to define the best methods for its assessment, as well as characterize and improve the treatment of ET dysfunctions.^1^, ^6^, ^9^, ^10^, ^11^, ^12^, ^13^, ^14^, ^15^, ^16^ Nevertheless, many unanswered questions remain, such as: what is the clinical utility of ETF tests in patients with intact TMs, and which is the optimal test protocol? Which level of change in middle ear pressure after performance of each test should be defined as the cutoff for positive or negative results, and what is the significance of a positive or negative finding in daily clinical practice? What is the utility of these tests in patients with pretest suspicion of ET dysfunction?

These doubts remain because, although several studies have assessed tubal function in ears with intact TMs by means of several tests, there is no consensus on standardization of test techniques. Comparison of results among these studies is therefore challenging, due to heterogeneity in test choice and administration, as well as measurement and reporting of results. Hence, strict testing and result reporting protocols are required.

The present study did not consider whether test results were positive or negative, as elsewhere in the literature, but rather if a statistically significant variation in mean pressures occurred. This criterion was chosen because, after an extensive review of the literature, it was determined that no consensus exists as to which change in pressure would constitute a positive result. Some studies advocate that any increase in pressure after the Valsalva maneuver be considered positive,^11^, ^15^, ^17^ whereas others adopt a cutoff point of 10 daPa.^1^, ^9^, ^18^ Establishing which level of change should be adopted to ensure clinical relevance is an extremely challenging proposition, as analysis of the difference in middle ear pressure that would lead to classification of a tympanogram as normal or abnormal showed enormous variation in pressure ranges (of up to 100 daPa) and demonstrated that the suggested cutoff point of 10 daPa is purely arbitrary.

With administration of the protocol proposed herein, the performance of consecutive Valsalva maneuvers led to an increase in mean pressures within the tympanic cavity in all three study groups at the first point of assessment. It was also found that the increase in pressure induced by the Valsalva maneuver was already significant after the first maneuver, and remained essentially stable with subsequent repetitions. Therefore, consecutive repetition of the Valsalva maneuver is not necessary to achieve an increase in pressure within the middle ear. Furthermore, even though a final increase in pressure occurred after the last of the consecutive maneuvers, ears in the moderate/severe retraction group continued to exhibit negative pressures after the complete sequence of five maneuvers, as corroborated by the presence of type C tympanograms. Thus it is questionable whether repeated maneuvers, as suggested by several authors for treatment of ET dysfunction, are really capable of improving ETF.^14^, ^16^

Analysis of the second time point of assessment showed a persistent trend toward increased middle ear pressures after the five consecutive maneuvers, although values did not reach statistical significance. It is believed that this is attributable to the wide variation in pressures in the moderate/severe TM retraction group.

Responses to the sniff test were distinct from responses to the Valsalva maneuver. In both the first and the second time points of assessment, the expected decrease in middle ear pressures failed to occur in any of the three groups of ears. Although response variability was greater in ears with moderate/severe TM retraction than in other groups, administration of the test maneuvers was not associated with significant changes in middle ear pressure. Similar responses were observed to the Toynbee maneuver, again both in the first and in the second time points of assessment: there were no significant changes in middle ear pressures from baseline after the five consecutive test maneuvers.

On comparison of mean pressures in each group of ears, it was found that these measurements were different from one another during all three tests, as patients with moderate/severe retraction had significantly more negative baseline pressures than the other patient groups. In the present sample, patients with mild TM retraction – despite near-normal responses to test maneuvers, as well as normal tympanograms in most cases – also had more negative baseline pressures than patients with healthy ears (all findings statistically significant).

Swarts et al.^17^ reported that 81% of patients were able to induce increases in middle ear pressure after a Valsalva maneuver. In a sample of healthy patients, Falk found that only 14% of the tested ears had negative pressure after the sniff test.^19^ A study of 32 patients found that only 5 (16%) exhibited changes in middle ear pressure after this test.^17^ Ryding et al.^20^ found that patients with a history of COM had significantly poorer active ETF as compared with healthy controls, and that ear with evidence of tubal dysfunction or patulous tubes had the most severe TM dysfunction. The present study corroborates the findings of these authors, demonstrating that the ears with the most severe TM changes exhibited the least response to Valsalva maneuvers. Bunne et al.^9^ also found the Valsalva maneuver to be more effective in healthy ears than after OM. Another study conducted by the same authors showed a 90% success rate for middle ear pressure equalization with this maneuver in healthy subjects, *vs.* only 48% in patients with TM retraction.^11^ In the same study, 26% of ears with TM retraction and 44% of healthy ears had a positive sniff test. Interestingly, in that study, retractions were not classified by severity, and, as in the present investigation, each degree of retraction was associated with a distinct pattern of response to tubal patency tests. Ears with mild retraction responded in a manner much closer to healthy ears than ears with marked TM retraction.

It is well known that the results of ETF tests may depend on how the test is performed (*e.g.*, how forcefully air is insufflated, and whether the patient swallows immediately after the maneuver). Although these variations may have occurred in the present study, particularly due to the lack of any increase in mean pressures in the moderate/severe retraction group after the Valsalva maneuver at time point 2, the findings are consistent with those reported in the literature, which suggests that the Valsalva maneuver is more effective than other tests.^11^ Another potential reason for the lack of any significant increase in pressure after the Valsalva maneuver in ears with more severe TM retraction is that these patients experience worse ear discomfort during the maneuver, which may lead to less forceful insufflation and, therefore, lessened effectiveness of the Valsalva test as compared to other patients.

In addition, only weak to moderate agreement was found between measurements obtained at the first and at the second time points of assessment with all three tests. This is consistent with the existing literature, and demonstrates substantial intra-individual variability.^9^ Furthermore, using all three tests, the range of difference between measurements obtained at the first and second time points was greater in ears with moderate/severe TM retraction, which shows that the behavior of these ears in response to ETF tests is even more unstable. This variability is consistent with the clinical instability often seen in patients with TM retraction and atelectasis.^16^, ^21^

In view of the broad variability in ETF tests, a single assessment of tubal opening is of limited applicability, particularly in patients with middle ear disease. ET opening and closure are less variable in normal ears than in ears with TM retraction.^11^, ^14^, ^16^, ^22^ If a test yields a positive result, ETF is probably good, but a negative result cannot be used to draw definitive conclusions about said function. According to Bunne et al.,^7^, ^11^ test results in a substantial proportion of patients shift from positive to negative or *vice versa* on re-administration of the Valsalva and sniff tests after 30 min. Falk and Magnuson reported similar findings regarding test instability, with qualitative responses to the sniff test changing in 30% of patients when retested on the same day.^14^

Therefore, ETF tests, which were widely employed in the past, are now of questionable clinical applicability, since their results are most variable and least reliable precisely in the patient population in whom they would be most indicated (ears with TM retraction and atelectasis).

## Conclusion

In the study population, mean pressures in the tympanic cavity tended to increase from baseline during the Valsalva maneuver, in all three study groups, only at the first time point of assessment. The expected changes in pressure during the sniff test and Toynbee maneuver did not occur in any of the three study groups at either time point of assessment. The normal ear and mild retraction groups behaved similarly between one another across all tests. The maneuvers studied herein exhibited weak to moderate intra-individual variation, demonstrating poor test replicability. Greater variation between measurements occurred among ears with moderate/severe TM retraction.

## Conflicts of interest

The authors declare no conflicts of interest.
